# Pathologic changes and immune responses against *Coxiella burnetii* in mice following infection via non-invasive intratracheal inoculation

**DOI:** 10.1371/journal.pone.0225671

**Published:** 2019-12-05

**Authors:** Xueyuan Hu, Yonghui Yu, Junxia Feng, Mengjiao Fu, Lupeng Dai, Zhiyu Lu, Wenbo Luo, Jinglin Wang, Dongsheng Zhou, Xiaolu Xiong, Bohai Wen, Baohua Zhao, Jun Jiao

**Affiliations:** 1 State Key Laboratory of Pathogen and Biosecurity, Beijing Institute of Microbiology and Epidemiology, Fengtai District, Beijing, China; 2 College of Life Sciences, Hebei Normal University, Yuhua District, Shijiazhuang, Hebei, China; University of Arkansas for Medical Sciences, UNITED STATES

## Abstract

Q fever is a worldwide zoonosis caused by *Coxiella burnetii*. Human Q fever is typically acquired through inhalation of contaminated aerosols, resulting in an initial pulmonary infection. In this study, BALB/c mice were infected with *C*. *burnetii* via an intratracheal (IT) route using a non-invasive aerosol pulmonary delivery device to directly place the living *C*. *burnetii* organisms into the lungs of the mice. The bacterial loads, pathological lesions, and antibody and cellular responses were analyzed and compared with those of mice infected via an intraperitoneal (IP) route. Compared with mice infected via an IP route, mice infected via an IT route exhibited a higher bacterial load and more severe pathological lesions in the heart and lungs at days 3 and 7 post-infection (pi). The levels of interferon-γ and IL-12p70 in the serum of mice infected via the IT route were significantly higher than those of mice infected via the IP route at day 3 pi. In conclusion, this murine model of acute *C*. *burnetii* infection via IT inoculation closely resembles the natural route of *C*. *burnetii* infection than that of IP injection. Thus, this newly developed model will be useful for investigating the pathogenesis and immunity of *C*. *burnetii* aerosol infection, as well as for the evaluation of therapeutic drugs and preventive vaccines of Q fever.

## Introduction

Q fever is a worldwide zoonosis caused by *Coxiella burnetii*, an intracellular Gram-negative bacterium. *C*. *burnetii* infection exhibits various acute and chronic clinical manifestations in humans. Acute Q fever is generally a flu-like illness with a high fever, headache, malaise, and myalgia [1], while chronic Q fever frequently presents as endocarditis [2], and/or hepatitis [3], and occasionally appears as osteomyelitis [4].

*C*. *burnetii* is able to infect a wide range of animals and livestock, with sheep, goats, and cattle as the major reservoirs [5, 6]. Most human acute *C*. *burnetii* infections occur after direct exposure to infected animals and their products, such as placenta, abortion products, wool, and manure. Occupations that require close contact with livestock, such as farmer, veterinarian, and abattoir worker, have a higher risk of acquiring Q fever [7–9]. Q fever has been recognized as an important infectious disease in many countries, including China. Furthermore, the Netherlands faced a very large outbreak of Q fever from 2007 to 2010, which demonstrated that Q fever has the potential to become a major public problem [10].

Animal models of acute *C*. *burnetii* infection usually employ mice, guinea pigs, or non-human primates [11], with murine models involving BALB/c and SCID strains being the most attractive animal models [12]. Aerosolization most closely resembles the natural route of *C*. *burnetii* infection in humans. Acute *C*. *burnetii* infection in mice may be caused by *C*. *burnetii* aerosolization using the whole-body aerosol exposure apparatus, but successful infection is dependent upon particle size and the anatomical parameters of the animal, and the procedure requires a higher titer of organisms in order to achieve study dosages within the lungs [13, 14]. In addition, the release of *C*. *burnetii* aerosol may cause abrupt infection in animals, and it remains uncertain if systemic dissemination of the infection comes from only the lungs or if it also involves extensive and complicated laboratory procedures [5, 15–17].

Another way to cause acute *C*. *burnetii* infection of mice in laboratories is to inoculate the organism directly into mice. Generally, there are three routes for the direct inoculation of *C*. *burnetii*: IP, IT, and intranasal (IN) [5, 18–21]. IP inoculation is a convenient route by which organisms quickly enter the circulation for systemic dissemination involving many organs, such as the heart, lungs, lymph nodes, and bone marrow of mice [21, 22], but it does not resemble the natural route of *C*. *burnetii* infection in humans. IN inoculation of *C*. *burnetii* closely resembles the natural route of *C*. *burnetii* infection in humans because the organisms may rapidly enter the lungs of the mice and cause pneumonia. However, only a minority of the *C*. *burnetii* inoculum is applied to the nares and respired through the nasal passages into the lungs of the mice during IN inoculation. The majority remains within the nasal passages [22, 23].

Unlike IN inoculation, IT inoculation more closely resembles the natural route of natural *C*. *burnetii* infection in humans. IT inoculation directly places a known quantity of the organism into the lungs of mice by using transoral intubation of trachea [24]. In the present study, a murine model of acute *C*. *burnetii* infection was developed using immunocompetent mice (BALB/c) via IT inoculation of *C*. *burnetii* using a non-invasive aerosol pulmonary delivery device. The bacterial loads, pathological lesions, serological and cellular responses were analyzed in mice and compared with those of mice infected via an IP route.

## Materials and methods

### Bacteria

*C*. *burnetii* Xinqiao strain (phase I virulent strain) was cultured in acidified citrate cysteine medium (ACCM)-2, passaged twice in a 2.5% oxygen environment, and then purified by high speed centrifugation as previously described [25]. The purified organisms were resuspended in phosphate-buffered saline buffer (PBS) and stored at -70°C.

### Animals

Specific-pathogen free (SPF) BALB/c mice (female, 6–8 weeks old) were purchased from Vital River Laboratories (Beijing, China). The mice were housed in sterile microisolator cages under a SPF environment in the animal biosafety level-3 (ABSL-3) laboratory.

### Infection

**Fig 1:**
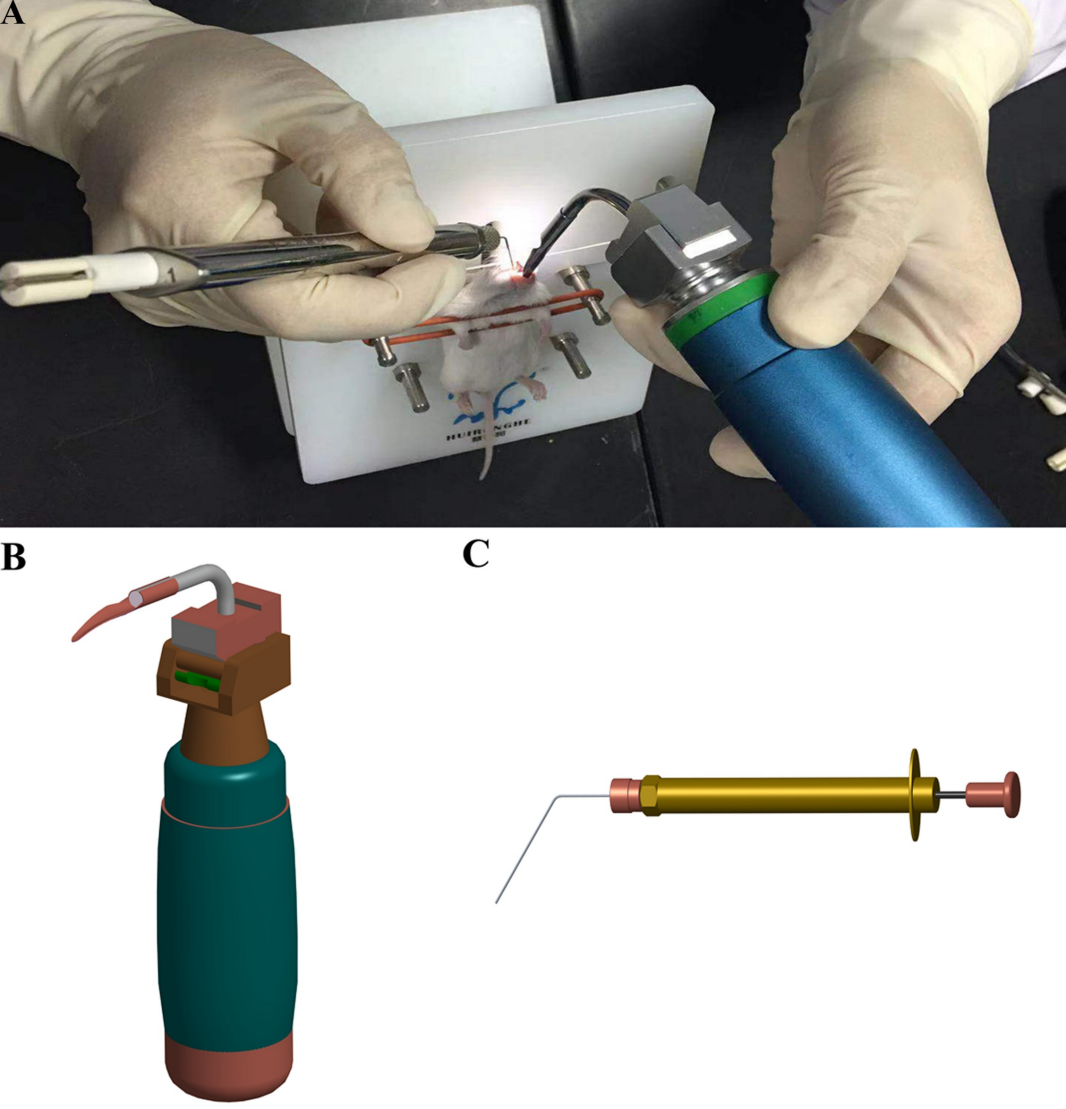
Schematic of mouse infection via a non-invasive intratracheal inoculation method. **A.** Representative image of non-invasive intratracheal inoculation. Anesthetized mice were placed on the slanted board. After a clear view of the trachea under the laryngoscope, the MicroSprayer Aerosolizer was inserted 25 mm from the larynx, and the suspension was sprayed. **B.** 3-D schematic of the laryngoscope. **C.** 3-D schematic of the MicroSprayer Aerosolizer.

All animal care and experimental procedures were in accordance with institutional policies for animal health and well-being and were approved by the Institutional Animal Care and Use Committee (IACUC) of the Academy of Military Medical Science (AMMS, Beijing, China). Mice (n = 12) were infected with 1 × 10^8^ *C*. *burnetii* organisms suspended in PBS (50 μl) via the IT route. The *C*. *burnetii* suspension was aerosolized directly into the lungs of BALB/c mice using the MicroSprayer Aerosolizer (Huironghe Company, Beijing, China) as previously described (Fig 1A) [26]. Briefly, mice were anesthetized by IP injection of pentobarbital sodium (100 mg/kg of body weight). Each mouse was then placed on a slanted board (60° angle from the horizontal direction) supported by a nylon band under its upper incisors and checked via the footpad reflex to confirm it was asleep. Once the trachea was in clear view under the laryngoscope (Fig 1B) (Huironghe Company, Beijing, China), the mouth cavity was lightened with shades of orange-red. The trachea then appeared as a white light spot, and then the MicroSprayer Aerosolizer (Fig 1C) was inserted 25 mm from the larynx (near the tracheal bifurcation), and 50 μl of the suspension was sprayed. Mice (n = 12) inoculated with PBS (50 μl) via the IT route were served as mock-inoculated control. In parallel, another group of mice (n = 12) were IP injected with 1 × 10^8^ *C*. *burnetii* organisms suspended in PBS (50 μl).

At days 3, 7, and 14 post-infection (pi), 4 mice in each group were anesthetized with pentobarbital sodium (100 mg/kg body weight) and euthanized via cervical dislocation. Blood was collected for serum isolation, and the bodies and spleens were weighed. The heart, liver, spleen, and lungs were also aseptically collected from every mouse. Each organ was divided into two parts—one part for the extraction of total genomic DNA for real-time quantitative polymerase chain reaction (qPCR) analysis and one part for histopathological analysis.

### qPCR specific for *C*. *burnetii*

Organ specimens (10 mg) and blood (100 μl) from each mouse were used to extract total genomic DNA using the DNeasy Blood & Tissue kit (Qiagen, GmbH, Germany) as described by the manufacturer. Each DNA sample was eluted from the DNA extraction column with elution buffer (200 μl). Each DNA sample (2 μl) was then used in a qPCR reaction with primers dotAF (5’-CCATGGCCCCAATTCTCTT-3’) and dotAR (5’-GCGCAATACGCTCAATCACA-3’) and a probe (5'6-FAM CCGGAGATACCGGCGGTGGG 3'TAMRA-N) targeting the *dotA* gene of *C*. *burnetii*.

### Histopathological analysis

Each organ specimen was fixed in 10% buffered-formalin for a minimum of 48 hours. The samples were then sectioned and embedded in paraffin. The paraffin-embedded tissue was sliced into thin sections to allow for adherence to the slides. The slides were then stained with hematoxylin and eosin (H&E) in order to observe pathological changes under light microscopy (BX60, Olympus, Japan). The histopathological findings (n = 10 high powered fields from each mice) were supported by pathological scores that evaluated by a trained pathologist according to the following scores: 0, not present; 1, mild; 2, moderate; 3, severe. The degree of pathological lesions was related to the distribution of lesions as follows: inflammatory cell infiltration, edema, congestion, and tissue necrosis.

### Detection of *Coxiella*-specific IgGs and cytokines in the sera of infected mice

IgGs to phase I or phase II antigens of *C*. *burnetii* were analyzed in the serum of each mouse via enzyme-linked immunosorbent assay (ELISA) in 96-well polystyrene plates (NUNC, Shanghai, China). The 96-well plates were coated with 100 μl of 1 × 10^8^ formalin-killed *C*. *burnetii* Xinqiao strain whole cells (phase I antigen) or Grita strain whole cells (phase II antigen) as described previously [27]. The cytokine levels, including interleukin-1β (IL-1β), interleukin-2 (IL-2), interleukin-4 (IL-4), interleukin-6 (IL-6), interleukin-10 (IL-10), interleukin-12p70 (IL-12p70), granulocyte-macrophage colony-stimulating factor (GM-CSF), interferon-γ (IFN-γ), and tumor necrosis factor-α (TNF-α), in the serum of each mouse were quantified by ProcartaPlex Immunoassays(Thermo, Beijing, China)using a Luminex Bio-Plex 200 IS 100 instrument (BIO-RAD, Hercules, CA, USA).

### Statistical analysis

The results of qPCR, specific IgG and cytokine levels, ratios of spleen to body weight and thickness of alveolar walls of the infected mice were expressed as the mean ± standard deviation (SD) of 4 mice per group. The differences in *C*. *burnetii* loads, specific IgG levels and cytokines levels in the sera, thickness of alveolar walls in the lungs and ratios of spleen to body weight were assayed using the two-way analysis of variance (ANOVA) test followed by the least significant difference (LSD) test, or Kruskal-Wallis test followed by Student-Newman-Keuls (SNK) test in Statistical Package in the Social Sciences version 19 software (SPSS, Chicago, IL, USA), according to the normality and homogeneity of variance of the data. *P* < 0.05 was considered significantly different.

## Results

### Clinical symptoms and *C*. *burnetii* dissemination in mice

**Fig 2:**
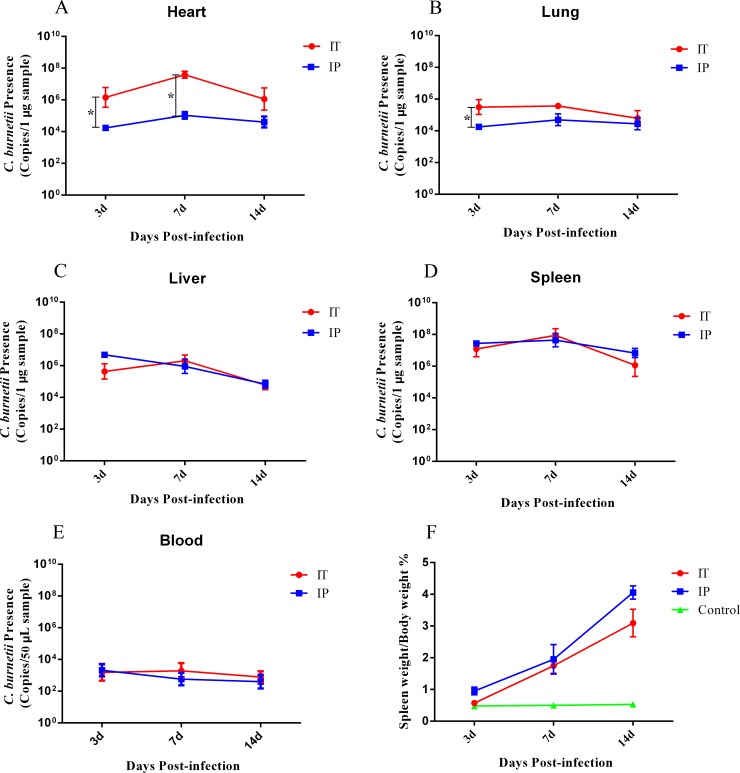
*C*. *burnetii* loads in organs and spleen/body weight of mice infected with *C*. *burnetii*, by IT route or IP route. Twelve BALB/c mice per group were infected with 1 × 10^8^ *C*. *burnetii* Xinqiao strain via an intratracheal route (IT) or intraperitoneal route (IP), or received an inoculum of 50 μl PBS via an IT route (Control). Four mice per group were euthanized at days 3, 7, and 14 pi. The differences in *C*. *burnetii* load and ratios of spleen to body weight were assayed using the two-way analysis of variance (ANOVA) test, followed by the least significant difference (LSD) test. *, *P*<0.05. **A**. The average *C*. *burnetii* load in the heart of mice from each group. **B**. The average *C*. *burnetii* load in the lungs of mice from each group. **C**. The average *C*. *burnetii* load in the liver of mice from each group. **D**. The average *C*. *burnetii* load in the spleen of mice from each group. **E**. The average *C*. *burnetii* load in the blood of mice from each group. **F**. The spleen/body weight ratio of each group of mice.

We monitored the development of symptoms and survival of the mice infected with *C*. *burnetii* by an IT and IP route. Approximately three days after infection with *C*. *burnetii*, all mice showed signs of illness, including ruffled coats and decreased activity, but none of the mice died. We then euthanized four mice per group at days 3, 7, and 14 pi, and the bacterial load in the blood, heart, lungs, liver, and spleen was detected by qPCR. The mouse bodies and spleens were also weighed. As shown in Fig 2, a large amount of *C*. *burnetii* was detected in the heart, lungs, liver and spleen of all the mice infected by both the IT and IP route at day 3 pi. The bacterial load in the heart, lungs, liver, and spleen at day 7 pi was higher than the bacterial load in these organs at days 3 or 14 pi. The bacterial load in the heart, but not in other organs of mice infected via the IT route, was significantly higher than that of the mice infected via the IP route at days 3 and 7 pi (Fig 2A–2E). The bacterial load in the lungs of mice infected via the IT route, was significantly higher than that of the mice infected via the IP route only at days 3 pi (Fig 2B). Low levels of bacterial load were detected in the blood of all the mice infected via both the IT and IP route (Fig 2E). In addition, the ratio of the spleen weight to the body weight of mice gradually increased during the infection course, but there was no significant difference between the IP-infected group and IT-infected group (Fig 2F).

### Histopathological findings

**Fig 3:**
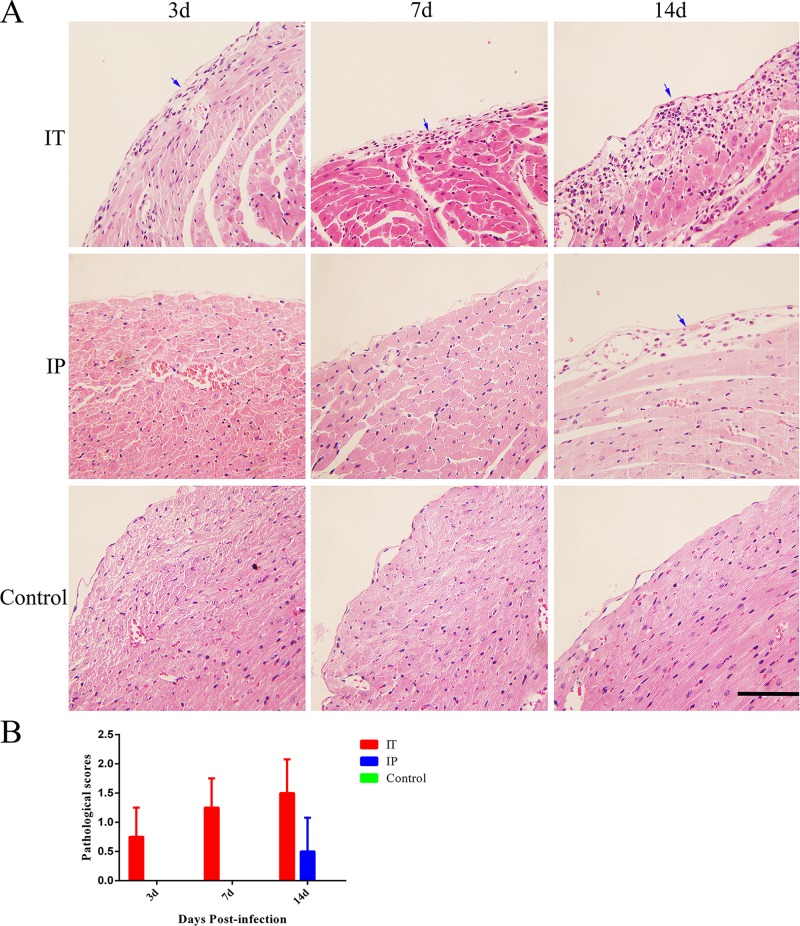
Histopathological analysis of cardiac tissue from mice infected with *C*. *burnetii* by an IT or IP route. BALB/c mice received an inoculum of 1 × 10^8^ *C*. *burnetii* Xinqiao strain via an intratracheal route (IT) or intraperitoneal route (IP), or received an inoculum of 50 μl PBS via an IT route (Control), and then the hearts were stained with hematoxylin and eosin (H&E). **A**. Histopathological lesions in the epicardium. **B**. The pathological scores of tissue sections after infection. A large number of inflammatory cells in the epicardium (shown with blue arrows) were observed in mice infected via the IT route at day 3 pi. A small number of inflammatory cells in the epicardium were noted in the mice infected via the IP route at day 14 pi. Original magnification, 400 ×. Scale bars, 100 μm.

**Fig 4:**
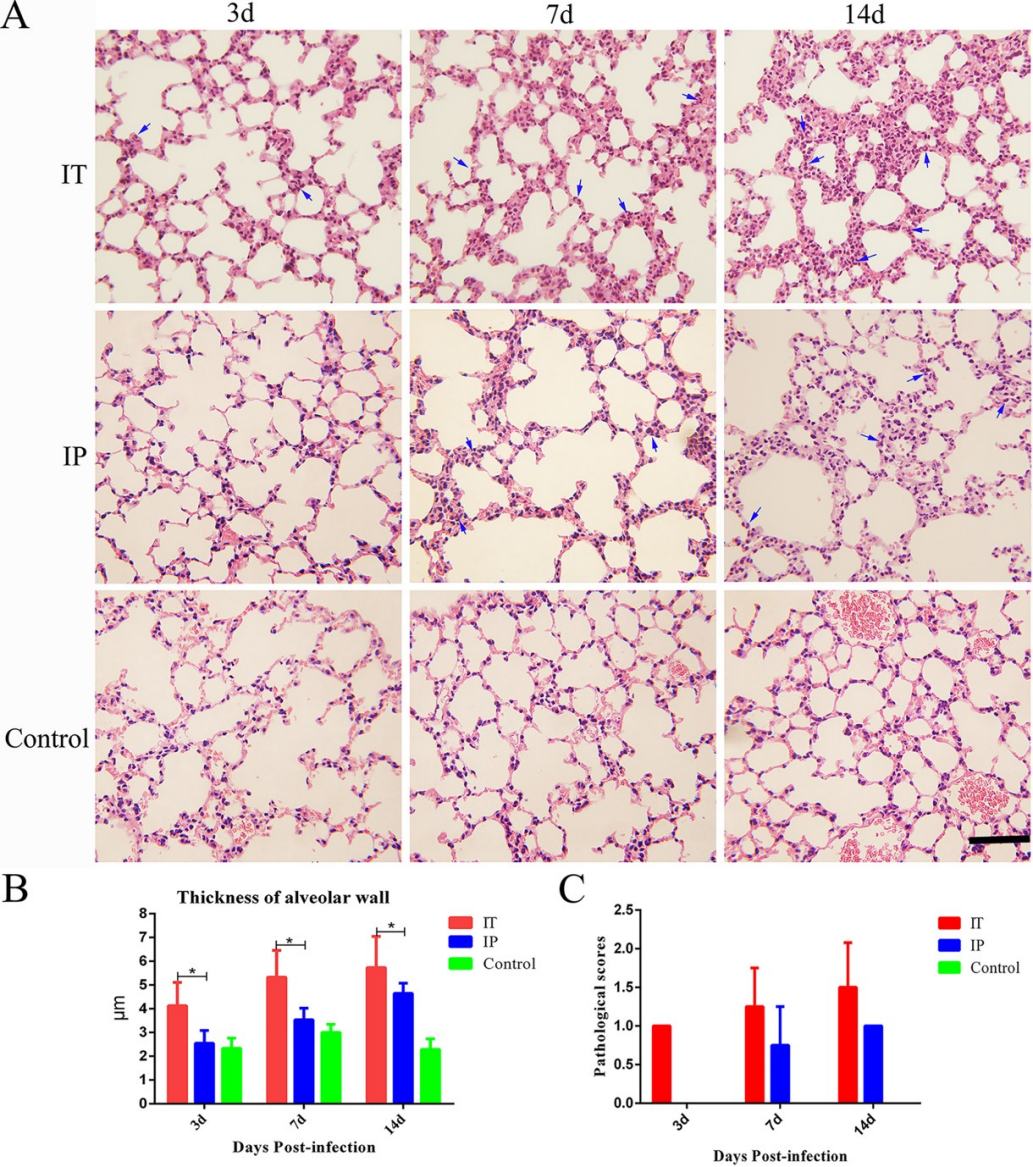
Histopathological analysis of the lungs of mice infected with *C*. *burnetii* by an IT or IP route. BALB/c mice received an inoculum of 1 × 10^8^ *C*. *burnetii* Xinqiao strain via an intratracheal route (IT) or intraperitoneal route (IP), or received an inoculum of 50 μl PBS via an IT route (Control), and then the lungs were stained with hematoxylin and eosin (H&E). The differences in the thickness of alveolar walls were assayed using the two-way analysis of variance (ANOVA) test followed by the least significant difference (LSD) test. *, *P*<0.05. **A**. Histopathological lesions in the lungs. **B**. Thickness of alveolar wall. **C**. The pathological scores of tissue sections after infection. The infiltration of inflammatory cells (shown with blue arrows) and increased thickness of alveolar walls were observed in the lungs of mice infected via the IT route at day 3 pi and in the lungs of mice infected via the IP route at day 7 pi. Original magnification, 400 ×. Scale bars, 100 μm.

In order to evaluate the histopathological lesions caused by *C*. *burnetii* infection via the IT route, we examined stained tissue sections from the heart, lungs, liver, and spleen of mice via light microscopy. Inflammatory cell infiltration were found in the epicardium (Fig 3) of all of the mice infected via the IT route at day 3 pi, and the number of inflammatory cells in the heart increased gradually at days 7 and 14 pi. In contrast, only a small number of inflammatory cells were found in the epicardium (Fig 3) of mice infected via the IP route at day 14 pi. No histopathological lesions were found in the heart of mice inoculated with PBS via the IT route (Fig 3). An infiltration of inflammatory cells including neutrophils, and increased thickness of alveolar wall were observed in the lungs of mice infected with *C*. *burnetii* via the IT route at days 3, 7, and 14 pi. Histopathological lesions in the lungs of mice infected via the IP route were not observed until day 7 pi (Fig 4). No histopathological lesions were observed in the lungs of mice inoculated with PBS via the IT route. The thickness of the alveolar walls in the lungs of mice infected with *C*. *burnetii* by the IT route was significantly higher than that of mice infected with *C*. *burnetii* via the IP route or that of mice inoculated with PBS via the IT route (Fig 4).

**Fig 5:**
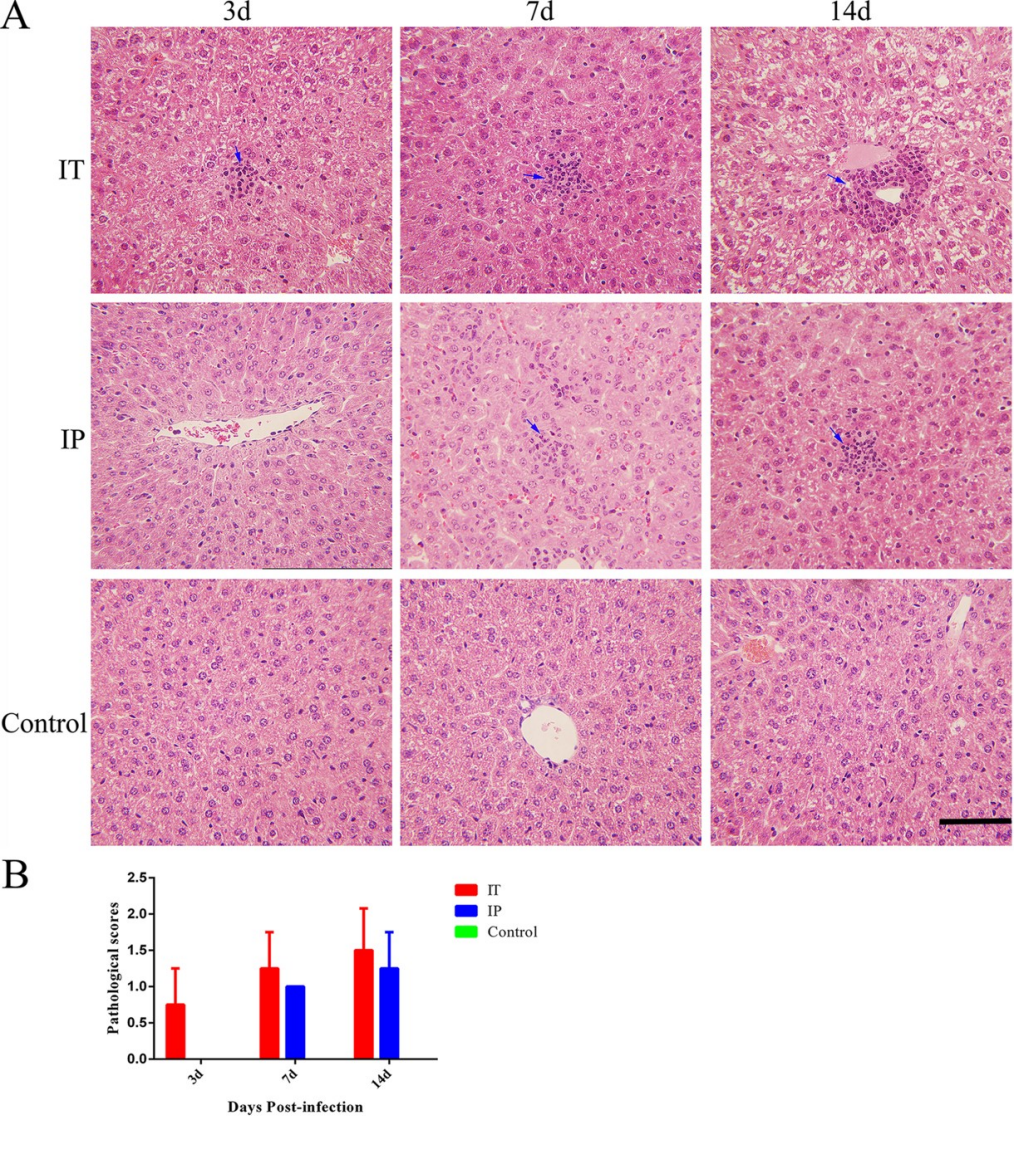
Histopathological analysis of the liver of mice infected with *C*. *burnetii* by an IT or IP route. BALB/c mice received an inoculum of 1 × 10^8^ *C*. *burnetii* Xinqiao strain via an intratracheal route (IT) or intraperitoneal route (IP), or received an inoculum of 50 μl PBS via an IT route (Control), and then the livers were stained with hematoxylin and eosin (H&E). **A**. Histopathological lesions in the liver. **B**. The pathological scores of tissue sections after infection. Small focal necrosis (shown with blue arrows) were observed in the livers of mice infected with *C*. *burnetii* via an IT route at day 3 or IP route at day 7 pi. Original magnification, 400 ×. Scale bars, 100 μm.

Small focal necrosis were also observed in the livers of mice infected with *C*. *burnetii* via the IT route at days 3, 7 and 14 pi, and these could be observed in the livers of mice infected with *C*. *burnetii* via the IP route at days 7 and 14 pi (Fig 5). The red pulp widened, the white pulp atrophied, and the splenic sinusoid extended with hyperemia in the spleens of mice infected with *C*. *burnetii* via the IT route from day 3 pi to day 14 pi (S1 Fig). Furthermore, the pathological changes in the liver and spleen of mice infected with *C*. *burnetii* via the IT route were comparable to that of mice infected via the IP route at day 7 and day 14 pi. In addition, no histopathological lesions were found in the liver and spleen of mice inoculated with PBS via the IT route.

### Serological and cellular responses against *C*. *burnetii* infection

**Fig 6:**
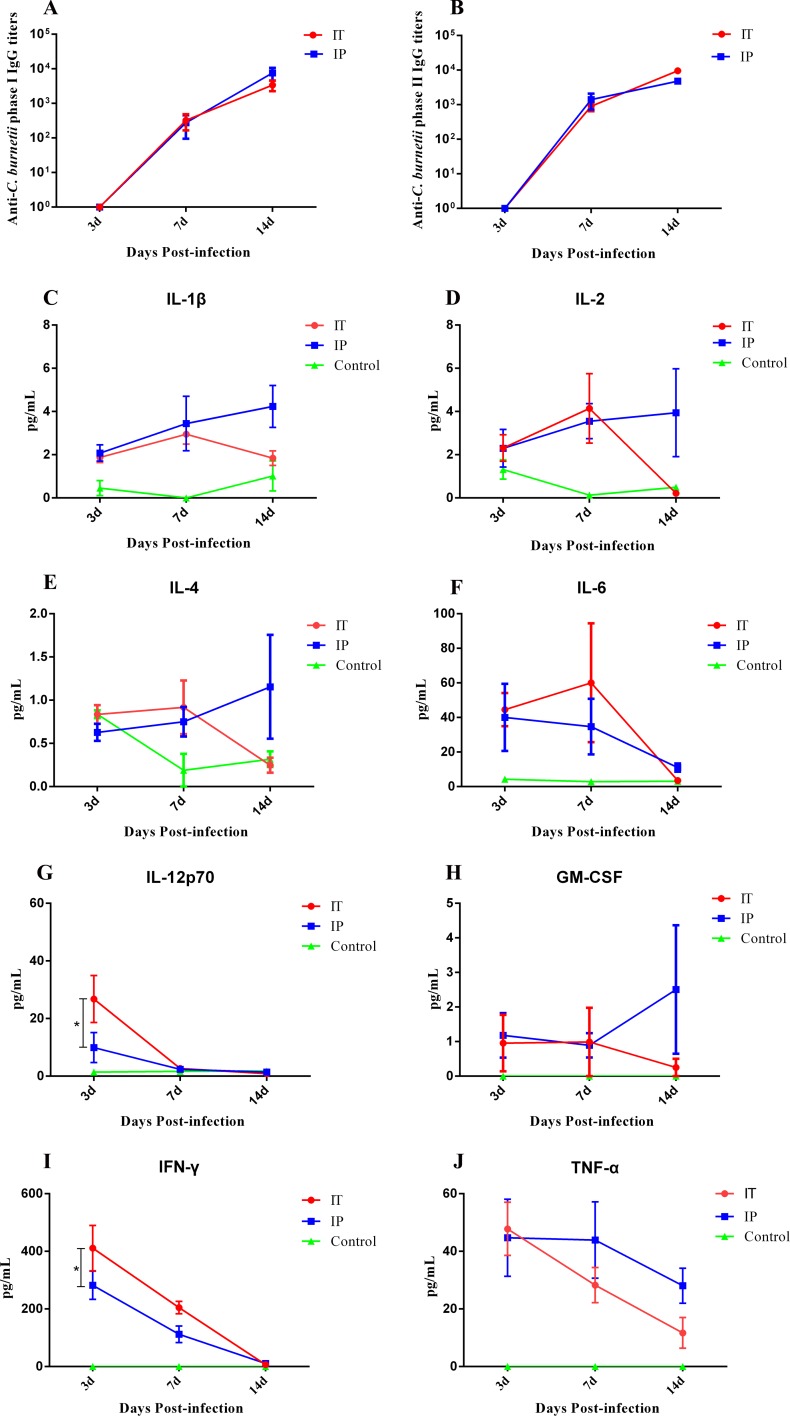
Antibody and cytokine analysis in the sera of mice infected with *C*. *burnetii* by an IT or IP route. BALB/c mice received an inoculum of 1 × 10^8^ *C*. *burnetii* Xinqiao strain via an intratracheal route (IT) or intraperitoneal route (IP), or received an inoculum of 50 μl PBS via an IT route (Control). Mice were euthanized at days 3, 7, and 14 pi. *C*. *burnetii*-specific IgG responses in the serum were measured by ELISA. Cytokines in the serum were quantified by ProcartaPlex Immunoassays. The differences in anti-*C*. *burnetii* IgG titers and cytokines levels in the sera were assayed using the two-way analysis of variance (ANOVA) test followed by the least significant difference (LSD) test, or the Kruskal- Wallis test followed by the Student-Newman-Keuls (SNK) test. *, *P*<0.05. **A**. The anti-*C*. *burnetii* phase I IgG titers during *C*. *burnetii* infection. **B.** The anti-*C*. *burnetii* phase II IgG titers during *C*. *burnetii* infection. **C**. The levels of IL-1β in the serum during *C*. *burnetii* infection. **D**. The levels of IL-2 in the serum during *C*. *burnetii* infection. **E**. The levels of IL-4 in the serum during *C*. *burnetii* infection. **F**. The levels of IL-6 in the serum during *C*. *burnetii* infection. **G**. The levels of IL-12p70 in the serum during *C*. *burnetii* infection. **H**. The levels of GM-CSF in the serum during *C*. *burnetii* infection. **I**. The levels of IFN-γ in the serum during *C*. *burnetii* infection. **J**. The levels of TNF-α in the serum during *C*. *burnetii* infection.

To better understand the murine humoral and cellular immune response against *C*. *burnetii* infection via the IT route, we determined the IgG levels to *C*. *burnetii* antigen by ELISA and the cytokine levels by multiplex immunoassay. As shown in Fig 6, no *C*. *burnetii*-special IgGs were detected at day 3 pi, the IgG antibody levels to phase I antigen (Fig 6A) and to phase II antigen (Fig 6B) in the serum of mice infected with *C*. *burnetii* via both the IT and IP route increased gradually throughout infection. The antibody levels to phase II antigen were higher than those to phase I antigen, indicating an acute stage of *C*. *burnetii* infection. The antibody levels of mice infected via the IT route were not significantly different from those of mice infected via the IP route at days 3, days 7 or days 14 pi. Increased levels of IL-6, IL-12p70, IFN-γ, and TNF-α were detected in the serum of IP-infected and IT-infected mice at day 3 pi, and the levels of those cytokines except IL-6 in the serum of IT-infected mice were decreased at days 7 and 14 dpi (Fig 6C–6J). The level of IL-12p70 and IFN-γ in the serum of mice infected via the IT route was significantly higher than that of mice infected via the IP route. Low levels of IL-1β, GM-CSF, IL-2, and IL-4 were detected in serum of mice infected via both the IT and IP route, but the levels were not significantly different. In addition, the production of IL-5 and IL-10 was undetectable in serum of mice infected via both the IT and IP route. In addition, the levels of cytokines of mice inoculated with PBS via the IT route were detected at low levels or undetectable.

## Discussion

Animal models of acute *C*. *burnetii* infection, which simulate the natural route of infection and clinical presentations associated with human acute Q fever, have been developed in guinea pigs, mice, and non-human primates. Mice, however, are the most attractive model to employ in *C*. *burnetii* infection [17, 28, 29]. Previous studies have shown that the aerosol inhalation procedure of infection in mice is relevant to the majority of acute Q fever cases in humans, and the clinical signs, bacterial burden, and pathological alterations in certain murine models may be used to monitor progression of the disease. The whole-body aerosol inhalation procedure was used in the mouse model of acute *C*. *burnetii* infection [12, 17, 30], but it may cause systemic dissemination of the infection in mice by other routes besides the respiratory tract. In the present study, the mouse model of acute *C*. *burnetii* infection was developed via an IT inoculation, which directly places the living *C*. *burnetii* into the lungs of immunocompetent BALB/C mice using a non-invasive aerosol pulmonary delivery device.

In this study, high bacterial loads were observed in all four organs at any time point pi, and the highest bacterial loads in these organs were determined at day 7 pi, suggesting that *C*. *burnetii* rapidly disseminated from the lungs to other organs. This results also demonstrated that *C*. *burnetii* grew stably and proliferated rapidly. Similar trends in bacterial loads were determined in the spleen of BALB/c mice infected by *C*. *burnetii* aerosolization [12]. The bacterial loads in these organs weren’t significantly different between day 3 pi and day 7 pi, which might be caused by high infection dose (1×10^8^ per mouse). Compared to other organs in mice infected with *C*. *burnetii* via the IT route, the bacterial load in the spleen was the highest, indicating that a larger amount of organisms disseminated into the spleens through the circulatory system, where the bacteria were engulfed by phagocytic cells in the spleen, which facilitated *C*. *burnetii* proliferation in the splenocytes [31]. In addition, the bacterial load in mice infected via the IT route at day 14 pi was lower than that at day 7 pi, suggesting that the immune system was activated to resist the infection, and the mice recovered in the late phase of the infection.

Pneumonia is a common complication during acute *C*. *burnetii* infection in humans because the lung is the earliest target organ in natural infection [32]. In the mice infected with *C*. *burnetii* via the IT route, the lungs were the foci of infection, and the pathological lesions in the lungs were more pronounced than those in the liver and spleen. Isolated hepatitis is a frequent presentation of acute *C*. *burnetii* infection in humans. Aside from granulomatous hepatitis and typical “doughnut” granulomas, atypical pathological changes were reported, such as epithelioid granuloma extensive extravasated fibrin and acute cholangitis without granuloma [33]. We observed small focal necrosis with infiltration of inflammatory cells in the liver of mice infected with *C*. *burnetii* via the IT route, suggesting that there were moderate pathological lesions in the liver.

Cases of pericarditis, acute myocarditis, and acute endocarditis were reported in acute *C*. *burnetii* infection in humans [34]. A large amount of *C*. *burnetii* organisms was observed in the heart of mice in the IT-infected group. Inflammatory cell infiltrates were observed in the epicardium of mice infected with *C*. *burnetii* via the IT route at day 3 pi, suggesting that early epicarditis and pathological lesions in the hearts of mice were compatible with heart diseases appearing during acute *C*. *burnetii* infection in humans. It is speculated that *C*. *burnetii* organisms arrived at the bronchi directly and rapidly reached the heart through the pulmonary aorta without the extensive circulatory system in mice infected with *C*. *burnetii* via the IT route. The pathological lesions were consistent with the bacterial load in the organs of the mice, confirming that the infection of *C*. *burnetii* disseminates from the lungs to other organs, such as the liver and spleen.

The levels of specific IgGs in mice infected with *C*. *burnetii* via the IT route were not significantly different from those in mice infected via the IP route, suggesting that the infection with *C*. *burnetii* via the IT route elicited specific immune responses against *C*. *burnetii*, similar to the IP route. IFN-γ and IL-12p70 are among the most important cytokines in the development of Th1 cells and initiation of cell-mediated immune responses [35, 36]. In this study, a significantly higher level of IL-12p70 and IFN-γ was detected during mice infection via the IP route at day 3 pi, which indicated that a greater cell-mediated immune response was efficiently induced in mice during the early stage of *C*. *burnetii* aerosol infection.

## Conclusions

In conclusion, the results in the present study confirm that a BALB/c murine model of acute *C*. *burnetii* infection was successfully established via IT inoculation with *C*. *burnetii* using a non-invasive aerosol pulmonary delivery device. *C*. *burnetii* was accurately delivered into the lungs of the mice. This modeled the pulmonary route of acute *C*. *burnetii* infection, which avoided potential confounding complications caused by other routes of infection, and simulated organ dissemination of the organism as seen during acute *C*. *burnetii* infection in humans. This newly developed model will be useful for investigating the pathogenesis and immunity of *C*. *burnetii* aerosol infection, as well as for the evaluation of therapeutic drugs and preventive vaccines of Q fever.

## Supporting information

S1 Fig Histopathological analysis of the spleens of mice infected with *C. burnetii* via an IT or IP route. BALB/c mice received an inoculum of 1 × 10^8^ *C*. *burnetii* Xinqiao strain via an intratracheal route (IT) or intraperitoneal route (IP), or received an inoculum of 50 μl PBS via an IT route (Control), and then the spleens were stained with hematoxylin and eosin (H&E). **A**. Histopathological lesions in the spleen. **B**. The pathological scores of tissue sections after infection. Original magnification, 200 ×. Scale bars, 100 μm.
(TIF)

S1 Data Bacterial loads and spleen to body weight after infection. (XLSX)

S2 Data Thickness of alveolar wall after infection. (XLSX)

S3 Data Antibody and cytokine analysis in the sera of infected mice. (XLSX)

S4 Data Pathological scores of organs after infection. (XLSX)

S1 File NC3Rs ARRIVE guidelines checklist. (DOCX)

S2 File Humane endpoints checklist. (DOCX)
