# Supplementary figures and images for: Pathologic changes and immune responses against Coxiella burnetii in mice following infection via non-invasive intratracheal inoculation
Source: PLoS One. 2019 Dec 5;14(12):e0225671. doi: 10.1371/journal.pone.0225671 (PMC6894818; doi:10.1371/journal.pone.0225671)

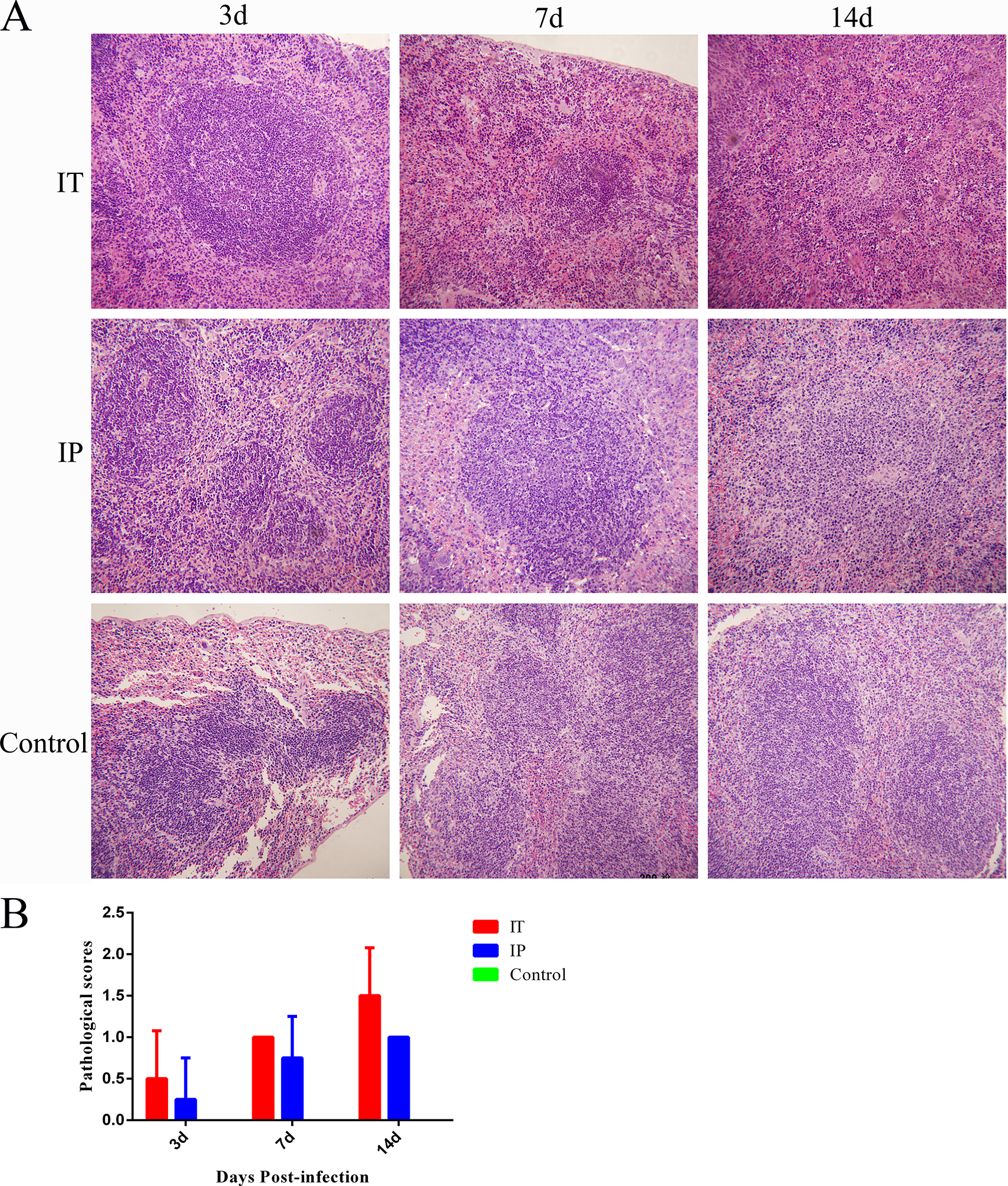

Supplement: S1 Fig — BALB/c mice received an inoculum of 1 × 10^8^ C. burnetii Xinqiao strain via an intratracheal route (IT) or intraperitoneal route (IP), or received an inoculum of 50 μl PBS via an IT route (Control), and then the spleens were stained with hematoxylin and eosin (H&E). A. Histopathological lesions in the spleen. B. The pathological scores of tissue sections after infection. Original magnification, 200 ×. Scale bars, 100 μm. (TIF) [file pone.0225671.s001.tif]
