# Supplementary material for: Pathologic changes and immune responses against Coxiella burnetii in mice following infection via non-invasive intratracheal inoculation
Source: PLoS One. 2019 Dec 5;14(12):e0225671. doi: 10.1371/journal.pone.0225671 (PMC6894818; doi:10.1371/journal.pone.0225671)

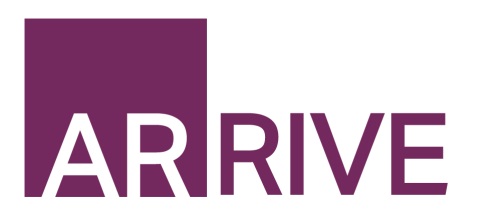


The ARRIVE Guidelines Checklist

Animal Research: Reporting In Vivo Experiments

Carol Kilkenny^1^, William J Browne^2^, Innes C Cuthill^3^, Michael Emerson^4^ and Douglas G Altman^5^

*^1^The National Centre for the Replacement, Refinement and Reduction of Animals in Research, London, UK, ^2^School of Veterinary Science, University of Bristol, Bristol, UK, ^3^School of Biological Sciences, University of Bristol, Bristol, UK, ^4^National Heart and Lung Institute, Imperial College London, UK, ^5^Centre for Statistics in Medicine, University of Oxford, Oxford, UK.*

|  | | ITEM | RECOMMENDATION | Section/ Paragraph |
| --- | --- | --- | --- | --- |
| 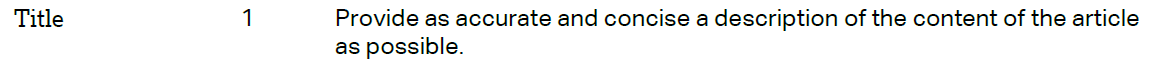 | | | 1 |  |
| 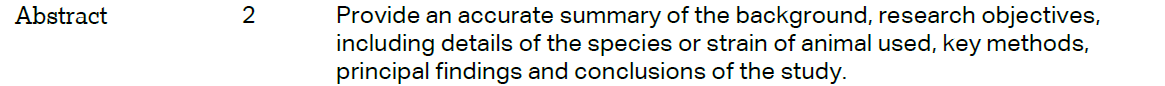 | | | 2 |  |
| INTRODUCTION | | |  |  |
| 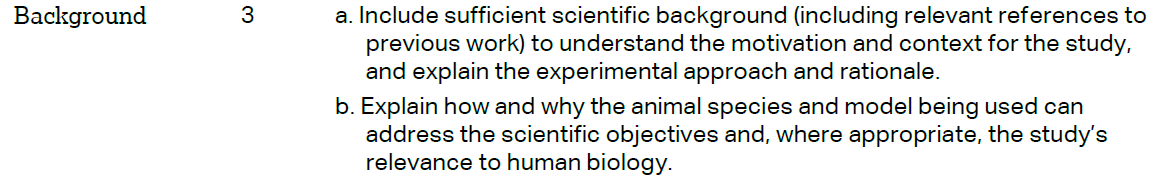 | | | 2-4 |  |
| 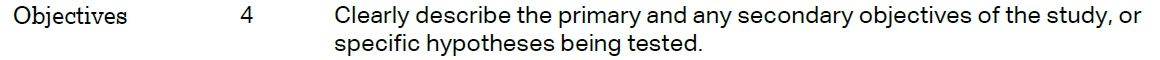 | | | 4 |  |
| METHODS | | |  |  |
| 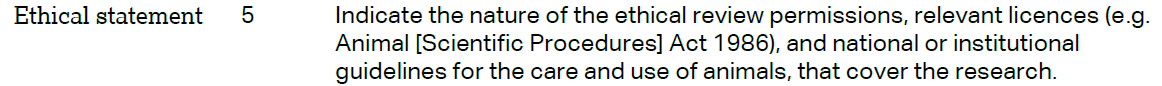 | | | 5, materials and methods, paragraph 3 |  |
| 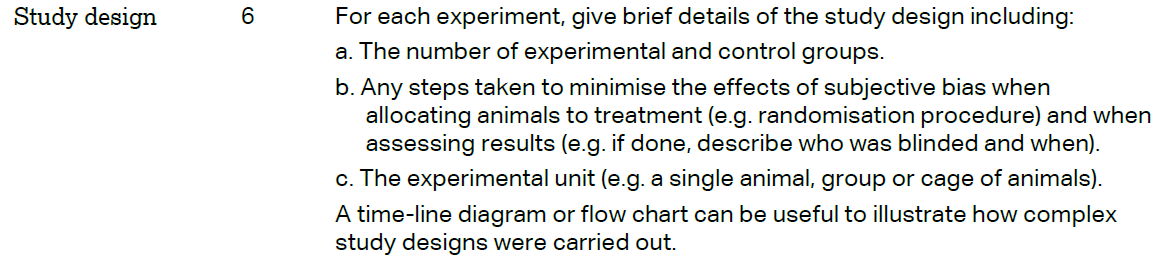 | | | 5 |  |
| 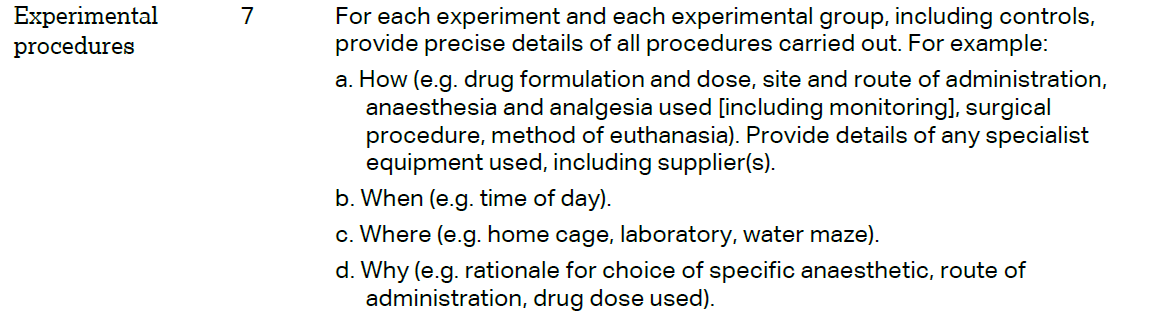 | | | 4-7 |  |
| 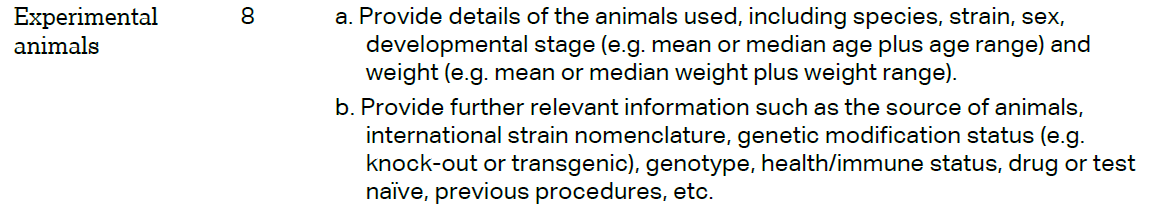 | | | 5, materials and methods, paragraph 2 |  |

The ARRIVE guidelines. Originally published in *PLoS Biology*, June 2010^1^

| 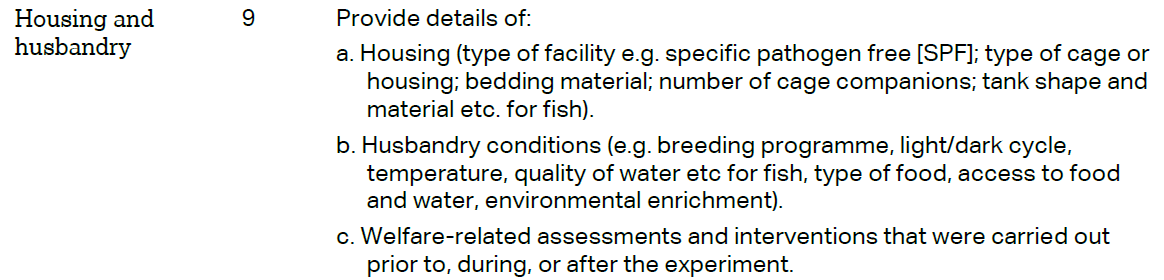 | 5, materials and methods, paragraph 2 | |
| --- | --- | --- |
| 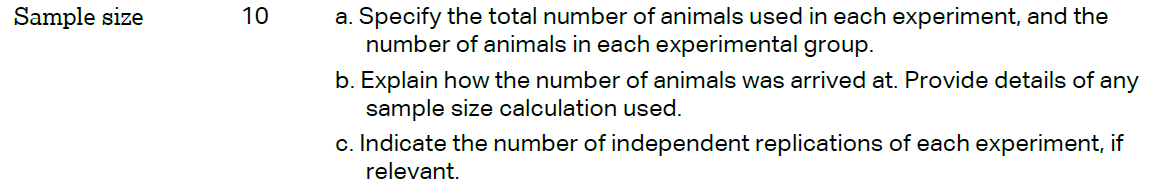 | 5, materials and methods, paragraph 3 | |
| 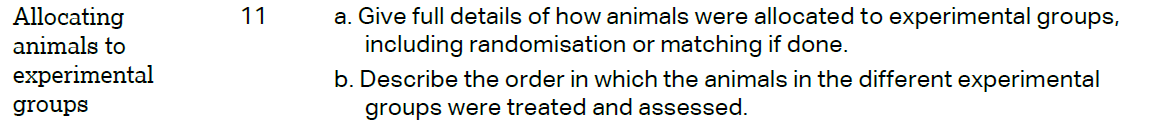 | 5, materials and methods, paragraph 3 | |
| 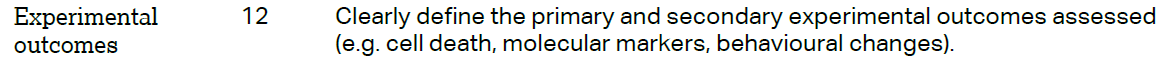 | 8-13 | |
| 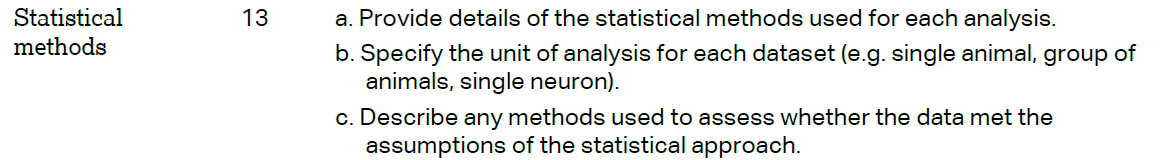 | 7-8, materials and methods, paragraph 8 | |
| RESULTS |  | |
| 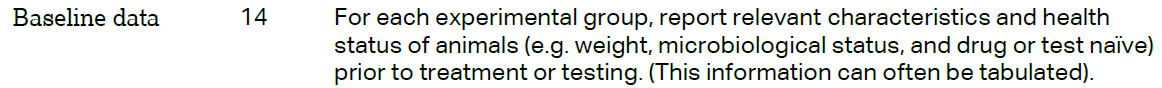 | N/A | |
| 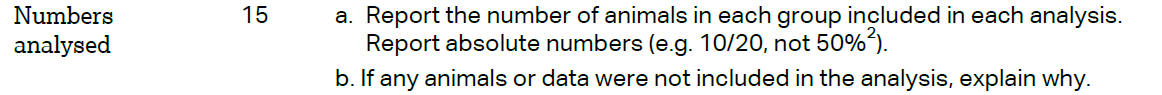 | 5 | |
| 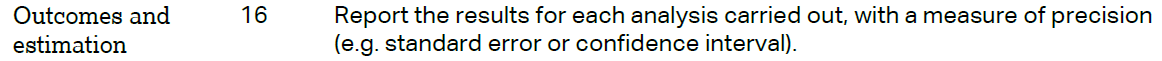 | N/A | |
| 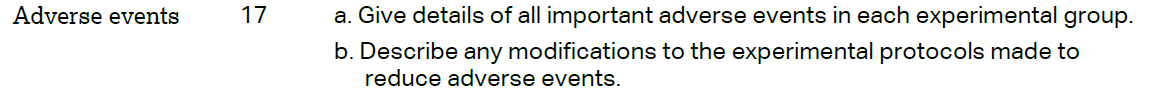 | N/A | |
| DISCUSSION |  | |
| 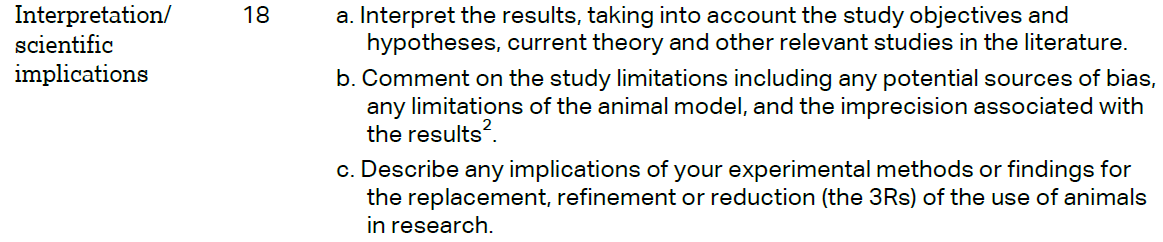 | 13-14 | |
| 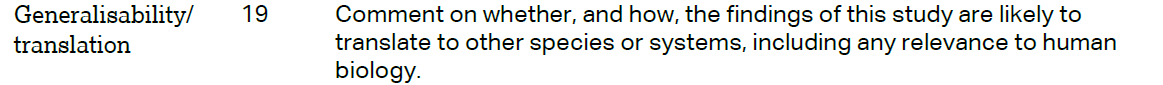 | N/A | |
| 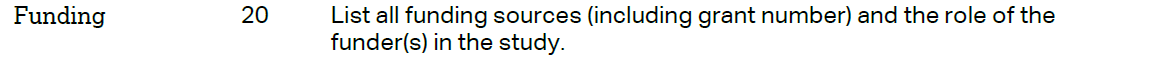 | | 17 |


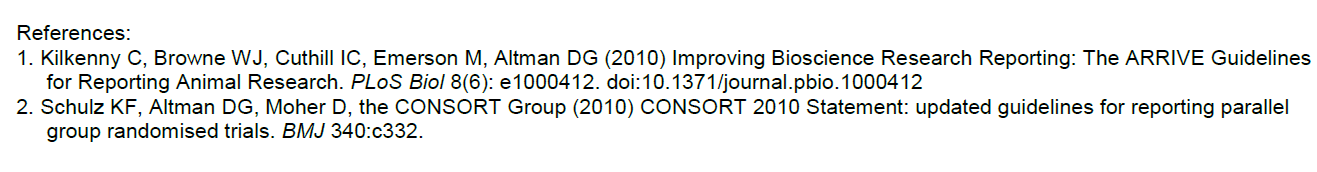

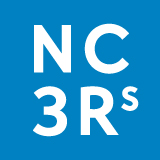

Supplement: S1 File — (DOCX) [file pone.0225671.s006.docx]
